# Socio-Economic and Clinical Factors as Predictors of Disease Evolution and Acute Events in COPD Patients

**DOI:** 10.1371/journal.pone.0135116

**Published:** 2015-08-07

**Authors:** Paolo Pandolfi, Alessandro Zanasi, Muriel Assunta Musti, Elisa Stivanello, Lara Pisani, Sabrina Angelini, Francesca Maffei, Silvana Hrelia, Cristina Angeloni, Corrado Zenesini, Patrizia Hrelia

**Affiliations:** 1 Department of Public Health, Local Health Authority of Bologna, Bologna, Italy; 2 Pneumology Unit, Sant’Orsola-Malpighi Hospital, Bologna, Italy; 3 Department of Clinical, Integrated and Experimental Medicine, Respiratory and Clinical Care Unit, Sant’Orsola-Malpighi Hospital, Bologna, Italy; 4 Department of Pharmacy and Biotechnology, Alma Mater Studiorum, University of Bologna, Bologna, Italy; 5 Department for Life Quality Studies, Alma Mater Studiorum, University of Bologna, Rimini, Italy; Johns Hopkins University, UNITED STATES

## Abstract

**Background:**

Socio-economic, cultural and environmental factors are becoming increasingly important determinants of chronic obstructive pulmonary disease (COPD). We conducted a study to investigate socio-demographic, lifestyle and clinical factors, and to assess their role as predictors of acute events (mortality or hospitalization for respiratory causes) in a group of COPD patients.

**Methods:**

Subjects were recruited among outpatients who were undertaking respiratory function tests at the Pneumology Unit of the Sant’Orsola-Malpighi Hospital, Bologna. Patients were classified according to the GOLD Guidelines.

**Results:**

229 patients with COPD were included in the study, 44 with Mild, 68 Moderate, 52 Severe and 65 Very Severe COPD (GOLD stage). Significant differences among COPD stage, in terms of smoking status and fragility index, were detected. COPD stage significantly affected the values of all clinical tests (spirometry and ABG analysis). Kaplan-Meier estimates showed a significant difference between survival curves by COPD stage with lower event-free probability in very severe COPD stage. Significant risk factors for acute events were: underweight (HR = 4.08; 95% CI 1.01–16.54), having two or more comorbidities (HR = 4.71; 95% CI 2.52–8.83), belonging to moderate (HR = 3.50; 95% CI 1.01–12.18) or very severe COPD stage (HR = 8.23; 95% CI 2.35–28.85).

**Conclusions:**

Our findings indicate that fragility is associated with COPD stage and that comorbidities and the low body mass index are predictors of mortality or hospitalization. Besides spirometric analyses, FeNO measure and comorbidities, body mass index could also be considered in the management and monitoring of COPD patients.

## Introduction

Chronic obstructive pulmonary disease (COPD) is a complex disease characterized by persistent airflow limitation, usually progressive, associated with enhanced chronic inflammatory response in the airways and the lung to noxious particles or gases [1]. COPD is a leading cause of morbidity and mortality worldwide, with peaks in particular in low and middle-income countries, and is responsible for an increase in social costs by governments and individuals.

The Global Burden of Disease Study estimated that COPD will become the third leading cause of death worldwide by 2020 [2]. In addition, considering the sum of years lost because of premature mortality and years of life lived with disability [Disability-Adjusted Life Year (DALY)], it has been estimated that by 2030 COPD will be the seventh leading cause of DALYs lost worldwide [2]. In Italy, COPD affects about 14% of the older population (65 years or more) and is the fifth cause of hospital admission in this age group. Several variables have been identified as factors influencing COPD life expectancy, including smoking, degree of dyspnoea, age, exercise capacity, body mass index (BMI), exacerbations, comorbidities, and quality of life [3–10]. They may be valuable in the assessment of severity and progression of disease and evaluate the response to medical intervention [11–12]. Several prognostic COPD indices have been identified [12]. However applying a prognostic index in a patient population other than the one in which it was developed, may require recalibration and/or modification [12].

At the base of major chronic diseases, such as COPD, there are common and modifiable risk factors, including unhealthy diet, tobacco use, alcohol abuse, and lack of physical activity but also non-modifiable risk factors such as age and genetic predisposition. In the last years, socio-economic, cultural, political and environmental factors are becoming increasingly important determinants of COPD. In view of this, we conducted a study to investigate socio-demographic, lifestyle and clinical factors in a population affected by COPD, and to assess their role as predictors of acute events (mortality or hospitalization for respiratory causes) in a group of COPD patients.

## Materials and Methods

### Subjects

The study was conducted as part of the multidisciplinary project *Respirare Bologna* (Breath Bologna) aimed at assessing determinants of health status outcomes in COPD patients.

Subjects were enrolled consecutively among outpatients who were undertaking respiratory function tests at the Pneumology Unit of the Sant’Orsola-Malpighi Hospital, Bologna, from October 2010 to July 2012. The only inclusion criterion was to be resident in Bologna, a city located in Northern Italy of about 380,000 inhabitants. The Pneumology Unit is the centre with the highest number of consultations within the four referral centres of the city, providing respiratory functions tests. Patients are usually sent by the General Practitioners for a first diagnosis of COPD and subsequent checks. COPD patients were classified according to GOLD guidelines [1] in: Mild (GOLD 1), Moderate (GOLD 2), Severe (GOLD 3) and Very Severe (GOLD 4).

### Ethics Statement

The present is an observational study where no new diagnostic tool and/or drug treatment was provided to any participant. Participants were treated according to routine clinical care. Likewise, patients data were collected as part of standard clinical care during a routine consultation. The authors were not involved in the patients medical treatment. According to the Italian law on retrospective evaluation of case series (Gazzetta Ufficiale n. 76, 31-3-2008) ethics approval was not necessary and authors did not ask for it. Nevertheless the study was conducted in accordance to the Italian Law n. 196/2003 about personal data treatment (D. Lgs 30 giugno 2003, n. 196. Gazzetta Ufficiale 2003, 174, S.O., 2003). Data were anonymized prior to the analyses after database linkage was done. Only one author conducting database linkage had access to patients identifying information. Only patients who provided written informed consent prior to participating in the study were enrolled according to the Helsinki Declaration and later Amendments. No minors/children were enrolled in the present study.

### Socio-demographic and clinical variables

Comprehensive socio-demographic, lifestyle and clinical data were collected by physician interviewers through the use of a predefined questionnaire during a routine clinical consultation. In particular, as far as socio-demographic and lifestyle characteristics, the following information were collected: age, gender, educational status, smoking status, including n. packs of cigarettes/year and physical activity (having carried out any physical activity that caused an increase in breathing, heart pulses or sweating). Moreover, for each participant a deprivation and a fragility index were attributed.

The deprivation index was developed by Caranci et al. using variables from the 2001 General Census of Population and Housing [13]. Five traits that represented the multidimensionality of the social and material deprivation concept were considered: low level of education, unemployment, non-home ownership, one-parent family and household overcrowding. The index is calculated by summing standardized indicators [13]. The fragility index, developed by the Local Health Authority of Bologna represents the probability of acute hospitalization or death in the following year and ranges from 0 to 100. The index was derived from a predictive model following the experience of the Combined Predictive Model [14] which aims to identify individuals at high-risk of re-hospitalization or death. The predictive model included demographic variables (age, gender), clinical variables such as heart failure, diabetes, cancer, lung disease, hospitalizations and access to emergency care during the previous year and social variables (deprivation index).

The following clinical characteristics were collected for each participant: Charlson index, Fraction Exhaled Nitric Oxide (FeNO), PaO2, PaCO2, pH, FVC, FEV1, FEV1/FVC (%), FVC (% of total) and FEV1 (% of total). The Charlson Comorbidity Index contains 19 categories of comorbidity [15]. In this paper this index is expressed in 3 categories: no comorbidity, one comorbidity and two or more comorbidities. FeNO levels were evaluated using a Niox chemiluminescence analyser (Aerocrine AB, Solna, Sweden). According to the American Thoracic Society (ATS) guideline, the subjects inhaled nitric oxide free air to total lung capacity and then exhaled at a constant flow rate against a valve connected to the nitric oxide analyzer [16]. We used the mean value of FeNO levels obtained in two tests. The measurements of pH, PaO_2_ and PaCO_2_ were evaluated using arterial blood gas (ABG) analysis. FEV1 and FVC were obtained by spirometry (Model N 403; Monaghan, Littleton, CO), with the spirometer calibrated daily. In addition, two anthropometric characteristics were collected: BMI and waist circumference. BMI was determined from height and weight measured at the time of the first visit, and categorized into four groups: underweight (<18.5kg/m^2^), normal (18.5–24.9kg/m^2^), overweight (25–29.9 kg/m^2^) and obese (>30kg/m^2^).

### Outcome

Acute events were defined in case of death for respiratory causes (X International Classification of Diseases codes J00-J99) or hospitalization for respiratory causes (IX International Classification of Diseases codes 460–519.9) occurred from October 2010 to December 2012 in and outside Bologna. Data were extracted from the Mortality Registry and the Hospital Admission Database of the Local Health Authority of Bologna.

### Statistical analysis

Continuous variables are presented as mean ± standard deviation (SD), while categorical variables as absolute frequency (relative frequency). Kruskal-Wallis, Pearson’s chi-square and Fisher’s exact tests were used to compare variables among COPD GOLD stages as appropriate. The unit of analysis was the patient. The survival function was calculated with Kaplan-Meier estimates for each GOLD stage and compared using log-rank test. Univariate and multivariate Cox regression analyses were performed to study the association between acute events and the following risk factors: age, gender, BMI, educational qualification, physical activity, smoking status, deprivation index, Charlson index and GOLD stages (Model 1). In a second model we replicated the analyses omitting the GOLD stage (Model 2). In a sensitivity analyses clinical variables such as PaO2 and FEV1 were considered instead of the GOLD stage. All *P*-values are based on 2-sided tests and *P* ≤ 0.05 were considered significant. Statistical analysis was performed using statistical package Stata Intercooled for Windows, version 12.0.

## Results

Table 1 shows the results regarding the social and lifestyles characteristics observed in the study population. 229 patients (129 male and 100 female; mean age 75±9.6 years) affected by COPD were enrolled in the study. According to the GOLD Guideline patients were classified as follows: 44 mild COPD, 68 moderate COPD, 52 severe COPD and 65 very severe COPD. In the overall population 42% of the patients had an education level of primary school, the majority (77.5%) did not practice any physical activity, more than 60% were ex-smokers and more than 50% turned out to be deprived or very deprived. The mean fragility index was 26.1. Significant differences within COPD stage, in terms of smoking status, were detected. Interestingly, a relationship between the increase of the fragility index and the severity of COPD was observed.

**Table 1:** Social and lifestyles characteristics in COPD population.

	COPD GOLD STAGE					
	Mild	Moderate	Severe	Very severe	Total	*P*-value
**N° subjects**	44	68	52	65	229	
**Age, mean**±**sd**	74.9±9.2	75.3±12.0	74.1±7.9	75.3±8.3	75.0±9.6	0.52
**Gender**						
Women	23 (52.3%)	32 (47.1%)	20 (38.5%)	25 (38.5%)	100 (43.7%)	
Men	21 (47.7%)	36 (52.9%)	32 (61.5%)	40 (61.5%)	129 (56.3%)	0.40
**Educational qualification**						
Middle school	10 (23.3%)	20 (29.4%)	13 (25.0%)	14 (22.2%)	57 (25.3%)	
No qualification	5 (11.6%)	3 (4.4%)	3 (5.8%)	6 (9.5%)	17 (7.5%)	
Primary school	16 (37.2%)	29 (42.7%)	20 (38.5%)	30 (47.6%)	95 (42.0%)	
Diploma	7 (16.3%)	10 (14.7%)	12 (23.1%)	9 (14.3%)	38 (16.8%)	
Degree	5 (11.6%)	6 (8.8%)	4 (7.7%)	4 (6.4%)	19 (8.4%)	0.89
**Physical activity**						
No	29 (72.5%)	49 (72.1%)	45 (86.5%)	46 (79.3%)	169 (77.5%)	
Yes	11 (27.5%)	19 (27.9%)	7 (13.5%)	12 (20.7%)	49 (22.5%)	0.23
**Smoking status**						
Never-smoker	15 (34.1%)	22 (32.4%)	7 (13.5%)	6 (9.4%)	50 (21.9%)	
Smoker	9 (20.5%)	7 (10.3%)	8 (15.4%)	8 (12.5%)	32 (14.0%)	
Ex-smoker	20 (45.4%)	39 (57.3%)	37 (71.2%)	50 (78.1%)	146 (64.1%)	0.0030
**Mean n. packs of cigarettes/year in smokers**±**sd**	28.1±15.5	45.1±79.2	40.1±23.3	44.1±19.8	40.7±43.7	0.0033
**Deprivation index**						
Very rich	9 (22.0%)	11 (16.9%)	9 (18.0%)	9 (14.8%)	38 (17.5%)	
Rich	8 (19.5%)	8 (12.3%)	4 (8.0%)	6 (9.8%)	26 (12.0%)	
Medium	3 (7.3%)	12 (18.5%)	10 (20.0%)	11 (18.0%)	36 (16.6%)	
Deprived	9 (21.9%)	13 (20.0%)	10 (20.0%)	9 (14.8%)	41 (18.9%)	
Very deprived	12 (29.3%)	21 (32.3%)	17 (34.0%)	26 (42.6%)	76 (35.0%)	0.73
**Fragility, mean**±**sd**	18.1±16.8	26.6±20.5	23.3±20.3	33.3±24.3	26.1±21.5	0.0024

Table 2 provides the details of clinical and anthropometric characteristics of the study population. There were non-significant differences in BMI and waist circumference among COPD stage. On the contrary, the stage was significantly associated with the values of all clinical tests (spirometry and ABG analysis). Furthermore the highest percentage of patients (42.2%) who had two or more comorbidities was observed in the group suffering from very severe COPD.

**Table 2:** Clinical and anthropometric characteristics in COPD population.

	COPD GOLD STAGE					
	Mild	Moderate	Severe	Very severe	Total	*P*-value
**BMI**						
Underweight	2 (4.6%)	0 (0.0)	3 (5.8%)	3 (4.7%)	8 (3.5%)	
Normal	17 (38.6%)	16 (23.9%)	17 (32.7%)	25 (39.1%)	75 (33.0%)	
Overweight	17 (38.6%)	32 (47.8%)	21 (40.4%)	22 (34.3%)	92 (40.5%)	
Obese	8 (18.2%)	19 (28.3%)	11 (21.1%)	14 (21.9%)	52 (22.9%)	0.34
**Waist circumference (cm)**	96.4±12.5	101.6±12.3	101.0±14.6	100.1±15.0	100.0±13.7	0.39
**Charlson index**						
No comorbidity	32 (72.7%)	33 (49.2%)	24 (46.2%)	20 (31.2%)	109 (48.0 &)	
One comorbidity	3 (6.8%)	16 (23.9%)	9 (17.3%)	17 (26.6%)	45 (19.8%)	
Two or more comorbidities	9 (20.5%)	18 (26.9%)	19 (36.5%)	27 (42.2%)	73 (32.2%)	0.0030
**FeNO (ppb)**	16.7±5.9	19.5±6.7	20.1±7.2	27.0±10.3	21.1±8.6	<0.001
**PaO2 (mmHg)**	79.7±8.9	73.8±10.0	69.9±10.1	71.1±14.8	73.1±12.2	<0.001
**PaCO2 (mmHg)**	38.3±4.1	40.4±5.6	41.8±6.9	49.7±10.5	43.0±8.7	<0.001
**pH**	7.44±0.03	7.43±0.03	7.44±0.08	7.42±0.04	7.43±0.05	0.048
**FVC (L)**	2.8±0.8	2.2±0.8	2.0±0.6	1.6±0.6	2.2±0.8	<0.001
**FEV1 (L)**	1.9±0.6	1.3±0.4	1.0±0.3	0.7±0.3	1.2±0.6	<0.001
**FEV1/FVC (%)**	67.6±6.6	59.9±11.1	51.9±12.9	45.5±11.8	55.6±13.6	<0.001
**FVC (% of total)**	102.9±19.5	81.3±14.5	69.6±16.3	57.1±17.8	76.0±23.2	<0.001
**FEV1 (% of total)**	87.8±13.5	61.4±7.5	44.8±9.6	32.8±12.2	54.7±22.3	<0.001

**Fig 1:**
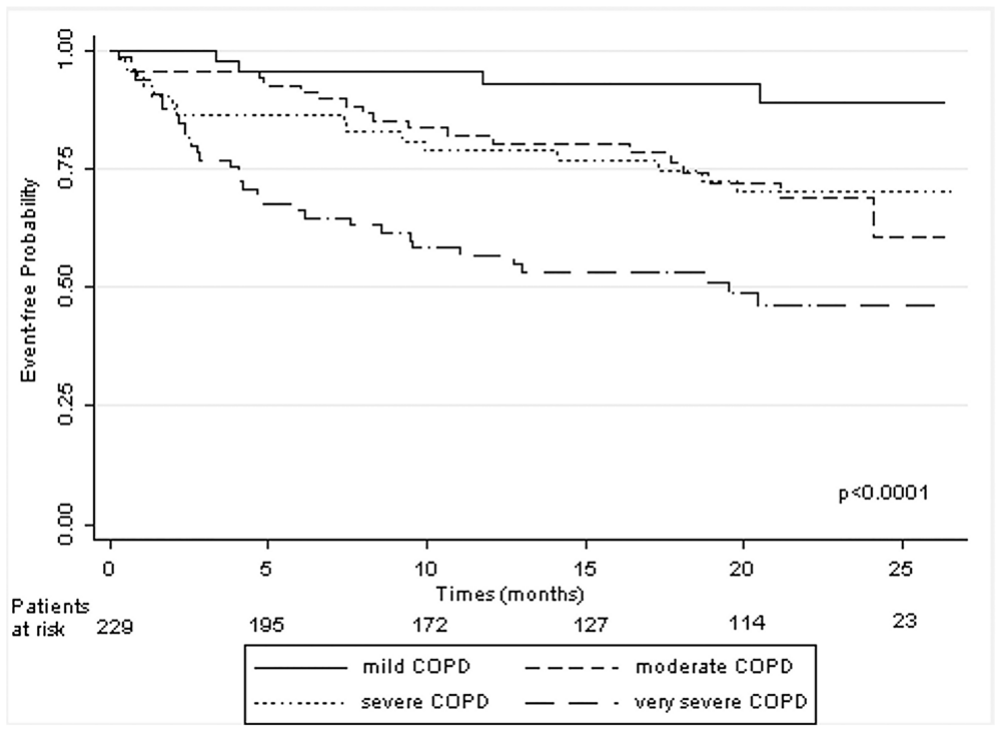
Kaplan-Meier analyses show that there is a significant difference between survival curves by COPD stage. The very severe COPD group has a lower event-free probability than subjects in the other stages. The mild COPD group has the highest event-free probability.

During the study period 71 patients experienced at least one acute event (69 hospitalizations and 14 deaths). Kaplan-Meier estimates show that there is a significant difference between survival curves by COPD stage (*P* < 0.001) (Fig 1).

In particular, by considering the single curves, patients in the mild group have a higher event-free probability than patients in the moderate group (*P* = 0.024), patients in the severe have a higher event-free probability than in the very severe group (*P* = 0.011) whereas in the moderate group patients do not show a significant difference in event-free probability compared with the severe group (*P* = 0.93).

Characteristics significantly associated with acute events in the univariate analyses were the Charlson index, COPD stage (Table 3) and FEV1(data not shown). In model 1, two or more comorbidities and COPD stage (mild and very severe) were confirmed as risk factors. Two or more comorbidities were associated with a 4.5-fold increased risk (HR = 4.50; 95% CI 2.39–8.49; *P* < 0.001); moderate and very severe COPD stages were associated with a 3.5 and a 8.23 –fold increased risk for acute events (HR = 3.50; 95% CI 1.01–12.18, *P* = 0.049 and HR = 8.23; 95% CI 2.35–28.85, *P* = 0.0010 respectively). As the GOLD stage could be on the causal pathway between some prognostic variables and COPD health status outcomes [17], we performed a multivariate analysis omitting GOLD stage as a covariate (Model 2). In this analysis underweight was a risk factors for acute events (HR = 4.08; 95% CI 1.01–16.54; *P* = 0.049). In the sensitivity analyses, when we included FEV1 and PaO_2_ instead of COPD stage in the model, gender, the Charlson index and FEV1 proved to be associated with acute events. A greater risk is observed in male gender vs female (HR 2.01; 95% CI 1.05–3.84), in patients with two or more comorbidities versus patients without comorbidities (HR = 4.17; 95% CI 2.16–8.04). A reduced risk is observed for every increase in 1 litre FEV1 (HR = 0.26; 95% CI 0.13–0.54) (S1 Table).

**Table 3:** Cox proportional hazards model of COPD population.

				Model 1		Model 2	
	Event	HR unadjusted (95% CI)	*P*-value	HR adjusted^a^ (95% CI)	*P*-value	HR adjusted^b^ (95% CI)	*P*-value
**Age**, mean±sd	76.33 ±8.48	1.02 (0.99–1.05)	0.25	1.00 (0.96–1.03)	0.80	1.00 (0.97–1.04)	0.88
**Gender**							
Women	29 (40.9%)	1.00		1.00		1.00	
Men	42 (59.2%)	1.19 (0.74–1.91)	0.48	1.33 (0.73–2.42)	0.35	1.27 (0.71–2.25)	0.42
**BMI**							
Normal	22 (31.4%)	1.00		1.00		1.00	
Underweight	3 (4.3%)	1.88 (0.56–6.31)	0.31	3.20 (0.73–14.11)	0.12	4.08 (1.01–16.54)	0.049
Overweight	29 (41.4%)	1.09 (0.62–1.89)	0.77	1.25 (0.66–2.36)	0.49	1.25 (0.67–2.34)	0.49
Obese	16 (22.9%)	1.01 (0.53–1.93)	0.96	0.87 (0.42–1.80)	0.71	0.87 (0.42–1.80)	0.70
**Educational qualification**							
Middle school	16 (22.9%)	1.00		1.00		1.00	
No qualification	6 (8.6%)	1.27 (0.50–3.24)	0.62	1.15 (0.40–3.32)	0.80	1.24 (0.46–3.39)	0.67
Primary school	31 (44.3%)	1.26 (0.69–2.31)	0.45	0.97 (0.50–1.88)	0.92	1.03 (0.53–2.03)	0.92
Diploma	10 (14.3%)	0.95 (0.43–2.09)	0.89	1.04 (0.44–2.45)	0.94	1.10 (0.48–2.51)	0.83
Degree	7 (10.0%)	1.26 (0.52–3.07)	0.61	1.41 (0.51–3.91)	0.51	1.32 (0.47–3.68)	0.60
**Physical activity**							
No	56 81.2%)	1.00		1.00		1.00	
Yes	13 (18.8%)	1.04 (0.56–1.91)	0.90	1.28 (0.62–2.62)	0.51	1.50 (0.73–3.07)	0.27
**Smoking status**							
Never-smoker	13 (18.3%)	1.00		1.00		1.00	
Smoker	8 (11.3%)	0.98 (0.40–2.36)	0.96	0.83 (0.28–2.41)	0.73	1.12 (0.39–3.23)	0.83
Ex-smoker	50 (70.4%)	1.48 (0.80–2.72)	0.21	0.94 (0.43–2.08)	0.89	1.39 (0.66–2.93)	0.39
**Charlson index**							
No comorbidity	17 (24.3%)	1.00		1.00		1.00	
One comorbidity	12 (17.1%)	1.76 (0.84–3.70)	0.13	1.27 (0.55–2.89)	0.58	1.74 (0.78–3.93)	0.18
Two or more comorbidities	41 (58.6%)	4.44 (2.52–7.82)	<0.001	4.50 (2.39–8.49)	<0.001	4.71 (2.52–8.83)	<0.001
**Deprivation index**							
Very rich	13 (19.1%)	1.00		1.00		1.00	
Rich	5 (7.4%)	0.50 (0.18–1.40)	0.19	0.51 (0.14–1.84)	0.31	0.49 (0.15–1.63)	0.25
Medium	11 (16.2%)	0.83 (0.37–1.85)	0.65	1.02(0.35–2.95)	0.97	1.35 (0.51–3.54)	0.55
Deprived	11 (16.2%)	0.65 (0.29–1.46)	0.30	0.72 (0.27–1.93)	0.51	0.89 (0.35–2.25)	0.80
Very deprived	28 (41.2%)	1.06 (0.55–2.05)	0.86	0.98 (0.36–2.67)	0.97	1.28 (0.52–3.14)	0.59
**COPD stage**							
Mild	4 (5.6%)	1.00		1.00		-	
Moderate	19 (26.8%)	3.21 (1.09–9.44)	0.034	3.50 (1.01–12.18)	0.049	-	
Severe	15 (21.1%)	3.22 (1.07–9.70)	0.038	2.87 (0.78–10.54)	0.11	-	
Very severe	33 (46.5%)	7.37 (2.61–20.82)	<0.001	8.23 (2.35–28.85)	0.0010	-	

^**a**^HR adjusted for age, gender, BMI, educational qualification, physical activity, smoking status, Charlson index, deprivation index and COPD stage

^**b**^HR adjusted for age, gender, BMI, educational qualification, physical activity, smoking status, Charlson index and deprivation index

## Discussion

In this study we investigated a panel of socio-demographic and clinical factors in a population affected by COPD, resident in Bologna. The overall study population consisted mostly of men, ex-smokers, not practicing any physical activity, obese or overweight. With regard to the deprivation index, the majority of patients were deprived or very deprived, regardless of COPD stage. Our findings are in line with previous literature’s results showing that smoking, aging, gender, and socio-economic factors are well established risk factors for COPD development [18]. Interestingly, taking into account the COPD GOLD stage, we observed a significant association between the fragility value, smoking status and the severity of the disease. Moreover we noted the highest percentage of patients with two or more comorbidities in patients suffering from very severe COPD. According to the literature, FeNO values are normal or mildly increased in stable COPD [19] and measurement of FeNO represents a non-invasive marker that may be useful to detect exacerbations and inflammation reduction in small airway disease [20]. In our study population FeNO values are associated with the severity of COPD. Given the type of study, we cannot deduce the direction of the association: frail subjects evolve more rapidly to very severe COPD stage but on reverse, COPD stage could increase the fragility of the patients.

Another objective of the present study was to identify predictors of acute events in terms of death or hospitalization in COPD patients. During the study period 71 patients had at last one acute event. Our findings indicate that subjects with very severe COPD or low FEV1 are at higher risk of death or hospitalization for respiratory causes compared to patients with mild COPD, confirming the validity of the spirometric test as prognostic factor. Our data confirm that patients classification according to the GOLD spirometric grading systems represents a predictor of exacerbations, hospitalizations and death [21–22].

COPD often coexists with other diseases and the scientific literature highlights that they may significantly impact on prognosis [1]. Our analyses confirms that the Charlson index is a risk factor for acute events, strengthening its role in COPD diagnosis and prognosis of poor outcomes. In addition, some studies highlighted a role of low BMI as an important risk factor for acute events, in particular showing that underweight and low skeletal muscle mass are significant determinants of mortality in COPD [23–27]. Our multivariate analysis provides results in line with the literature suggesting that recording of weight should be part of the follow up of these patients. The heterogeneous distribution of underweight among patients with different characteristics (e.g. deprivation) and the sample size may explain the non significant results of BMI as a risk factor in the bivariate analyses.

Previous studies have examined the association between socio-economic status and COPD health outcomes but results are controversial, possibly due to the different accessibility to health care [28–31]. Eisner et al. [17] found that socio-economic status represents a risk factor for adverse COPD health outcomes; in contrast, in our study the deprivation index does not influence the incidence of acute events. Differences between the US and the Italian health care system could explain the different results between the two studies. In particular, the access to the national health service guaranteed to the entire population might mitigate the effect of deprivation.

Our study measured only respiratory-associated events. Deaths in individuals with COPD are frequently attributed to a cause other than respiratory such as cardiovascular disease or other causes [18]. Our results are therefore not directly generalizable to events for other causes. Indeed, this could represent a limitation of our study, however the main aim has been to assess the efficacy of a panel of life-style and clinical factors as predictors of acute events, including mortality and/or hospitalization, for respiratory causes. Another limitation is the small sample size, consequently we cannot exclude that predictors investigated in the present study might result significantly associated with the outcomes under investigation in larger studies.

In conclusion, a relationship between the increase of the fragility index and the severity of COPD was observed. Moreover, our findings indicate that the Charlson index is associated with disease evolution and that it could be considered as a risk factor for acute events. On the other hand, the deprivation index does not influence both disease evolution and acute events, probably because the accessibility to the Italian National Health Service, countervails the effects associated to socio-economic status. Furthermore, besides spirometric analyses and FeNO measure, our findings suggest that BMI could be considered in the management and monitoring of COPD patients.

## Supporting Information

S1 Database (XLS)

S1 Table Cox proportional hazards model of COPD population. (DOC)

## References

[1] Roisin RR, Anzueto A, Bourbeau J, deGuia TS, Hui DSC, Martinez F et al Global Strategy for the Diagnosis, Management and Prevention of COPD, Global Initiative for Chronic Obstructive Lung Disease (GOLD) 2010.

[2] Mathers CD, Loncar D. Projections of global mortality and burden of disease from 2002 to 2030. PLoS Med 2006; 3:e442 17132052 10.1371/journal.pmed.0030442 PMC1664601

[3] Scognamiglio A, Matteelli G, Pistelli F, Baldacci S, Carrozzi L, Viegi G. L’epidemiologia della broncopneumopatia cronica ostruttiva. Ann Ist Super Sanità 2003; 39(4):467–484. 15098569

[4] Ferrer M, Alonso J, Morera J, Marrades RM, Khalaf A, Aguar MC et al Chronic obstructive pulmonary disease stage and health-related quality of life. Ann Intern Med 1997; 127:1072–9. 9412309 10.7326/0003-4819-127-12-199712150-00003

[5] Jones PW. Health status measurement in chronic obstructive pulmonary disease. Thorax 2001; 56:880–7. 11641515 10.1136/thorax.56.11.880 PMC1745959

[6] Nishimura K, Izumi T, Tsukino M, Oga T. Dyspnea is a better predictor of 5-year survival than airway obstruction in patients with COPD. Chest 2002; 121:1434–40. 12006425 10.1378/chest.121.5.1434

[7] Soler-Cataluña JJ, Martínez-García MA, Román Sánchez P, Salcedo E, Navarro M, Ochando R. Severe acute exacerbations and mortality in patients with chronic obstructive pulmonary disease. Thorax 2005; 60(11):925–31. 16055622 10.1136/thx.2005.040527 PMC1747235

[8] Mannino DM, Thorn D, Swensen A, Holguin F. Prevalence and outcomes of diabetes, hypertension and cardiovascular disease in COPD. Eur Respir J 2008; 32:962–9. 10.1183/09031936.00012408 18579551

[9] Landbo C, Prescott E, Lange P, Vestbo J, Almdal TP. Prognostic Value of Nutritional Status in Chronic Obstructive Pulmonary Disease. Am J Respir Crit Care Med 1999; 160:1856–61. 10588597 10.1164/ajrccm.160.6.9902115

[10] Eisner MD, Anthonisen N, Coultas D, Kuenzli N, Perez-Padilla R, Postma D et al Committee on Nonsmoking COPD, Environmental and Occupational Health Assembly. An Official American Thoracic Society Public Policy Statement: Novel Risk Factors and the Global Burden of Chronic Obstructive Pulmonary Disease. Am J Respir Crit Care Med 2010; 182:693–718. 10.1164/rccm.200811-1757ST 20802169

[11] Celli BR. Predictors of mortality in COPD. Respir Med 2010; 104(6):773–9. 10.1016/j.rmed.2009.12.017 20417082

[12] Dijk WD, Bemt L, Haak-Rongen S, Bischoff E, Weel CV, Veen JC et al Multidimensional prognostic indices for use in COPD patient care. A systematic review. Respir Res 2011; 2:151 10.1186/1465-9921-12-151 PMC3228786 22082049

[13] Caranci N, Biggeri A, Grisotto L, Pacelli B, Spadea T, Costa G. The Italian deprivation index at census block level: definition, description and association with general mortality. Epidemiol Prev 2010; 34(4):167–76. 21224518

[14] Wennberg D, Siegel M, Darin B, Filipova N, Russell R, Kenney L et al Combined predictive model Final report & technical documentation. Cambridge, UK: HealthDialog UK Limited, King’s Fund, New York University 2006.

[15] Charlson ME, Pompei P, Ales KL, MacKenzie CR. A new method of classifying prognostic comorbidity in longitudinal studies: development and validation. J Chronic Dis 1987; 40:373–83. 3558716 10.1016/0021-9681(87)90171-8

[16] Dweik RA, Boggs PB, Erzurum SC, Irvin CG, Leight MW, Lundberg JO et al An official ATS clinical practice guideline: interpretation of exhaled nitric oxide levels (FENO) for clinical applications. Am J Respir Crit Care Med 2011; 184:602–615. 10.1164/rccm.9120-11ST 21885636 PMC4408724

[17] Eisner MD, Blanc PD, Omachi TA, Yelin EH, Sidney S, Katz PP et al Socioeconomic status, race and COPD health outcomes. J Epidemiol Community Health 2011; 65(1):26–34. 10.1136/jech.2009.089722 19854747 PMC3017471

[18] Mannino DM, Buist AS. Global burden of COPD: risk factors, prevalence, and future trends. Lancet 2007; 370(9589):765–73. 17765526 10.1016/S0140-6736(07)61380-4

[19] Taylor DR, Pijnenburg MW, Smith AD, Jongste JCD. Exhaled nitric oxide measurements: clinical application and interpretation. Thorax 2006; 61:817e27. 16936238 10.1136/thx.2005.056093 PMC2117092

[20] Papi A, Romagnoli M, Baraldo S, Braccioni F, Guzzinati I, Saetta M, et al Partial reversibility of airflow limitation and increased exhaled NO and sputum eosinophilia in chronic obstructive pulmonary disease. Am J Respir Crit Care Med 2000; 162(5):1773–1777. 11069811 10.1164/ajrccm.162.5.9910112

[21] Revill SM, Morgan MD, Singh SJ, Williams J, Hardman AE. The endurance shuttle walk: a new field test for the assessment of endurance capacity inchronic obstructive pulmonary disease. Thorax 1999; 54(3):213–22. 10325896 10.1136/thx.54.3.213 PMC1745445

[22] Agusti A, Calverlev PM, Celli B, COxson HO, Edwards LD, Lomas DA et al Characterisation of COPD heterogeneity in the ECLIPSE cohort. Respir Res 2010; 11:122 20831787 10.1186/1465-9921-11-122 PMC2944278

[23] Schols AM, Broekhuizen R, Weling-Scheepers CA, Wouters EF. Body composition and mortality in chronic obstructive pulmonary disease. Am J Clin Nutr 2005; 82(1):53–9. 16002800 10.1093/ajcn.82.1.53

[24] Vestbo J, Prescott E, Almdal T, Dahl M, Nordestgaard BG, Andersen T et al Body mass, fat-free body mass, and prognosis in patients with chronic obstructive pulmonary disease from a random population sample: findings from the Copenhagen City Heart Study. Am J Respir Crit Care Med 2006; 173(1):79–83. 16368793 10.1164/rccm.200506-969OC

[25] Rutten E, Calverley P, Casaburi R, Agusti A, Bakke P, Celli B et al Changes in Body Composition in Patients with Chronic Obstructive Pulmonary Disease: Do They Influence Patient-Related Outcomes?. Ann Nutr MEtab 2013; 63:239–247 10.1159/000353211 24216978

[26] Zhou Y, Wang D, Liu S, Lu J, Zheng J, Zhong N et al The association between BMI and COPD: the results of two population-based studies in Guangzhou, China. COPD 2013; 10(5):567–72. 10.3109/15412555.2013.781579. Epub 2013 Jul 11. 23844907

[27] Cazzola M, Calzetta L, Lauro D, Bettoncelli G, Cricelli C, Di Daniele N et al Asthma and COPD in an Italian adult population: role of BMI considering the smoking habit. Respir Med 2013; 107(9):1417–22. 10.1016/j.rmed.2013.04.021 23702090

[28] Ketelaars CA, Schlösser MA, Mostert R, Huyer Abu-Saad H, Halfens RJ, Wouters EF. Determinants of health-related quality of life in patients with chronic obstructive pulmonary disease. Thorax 1996; 51:39–43. 10.1136/thx.51.1.39 8658367 PMC472797

[29] Afessa B, Morales IJ, Scanlon PD, Peters SG. Prognostic factors, clinical course, and hospital outcome of patients with chronic obstructive pulmonary disease admitted to an intensive care unit for acute respiratory failure. Crit Care med 2002; 30(7):1610–5. 12130987 10.1097/00003246-200207000-00035

[30] Prescott E, Godtfredsen N, Vestbo J, Osler M. Social position and mortality from respiratory diseases in males and females. Eur Respir J 2003; 21(5):821–6. 12765428 10.1183/09031936.03.00047502

[31] Power C, Hypponen E, Smith GD. Socioeconomic position in childhood and early adult life and risk of mortality: a prospective study of the mothers of the 1958 British birth cohort. Am J Public Health 2005; 95:1396–402. 15985645 10.2105/AJPH.2004.047340 PMC1449372

