# Supplementary material for: Socio-Economic and Clinical Factors as Predictors of Disease Evolution and Acute Events in COPD Patients
Source: PLoS One. 2015 Aug 7;10(8):e0135116. doi: 10.1371/journal.pone.0135116 (PMC4529271; doi:10.1371/journal.pone.0135116)
Supplement: S1 Table — (DOC) [file pone.0135116.s002.doc]

**S1 Table. Cox proportional hazards model of COPD population.**

|  | **Event** | **HR adjusted^a^**  **(95% CI)** | ***P* -value** |
| --- | --- | --- | --- |
|
|
| **Age**, mean±sd | 76.33 ±8.48 | 0.99 (0.95-1.02) | 0.42 |
| **Gender** |  |  |  |
| Women | 29 (40.9 %) | 1.00 |  |
| Men | 42 (59.2 %) | 2.01 (1.05-3.84) | 0.034 |
| **BMI** |  |  |  |
| Normal | 22 (31.4 %) | 1.00 |  |
| Underweight | 3 (4.3 %) | 1.80 (0.39-8.30) | 0.45 |
| Overweight | 29 (41.4 %) | 1.14 (0.60-2.15) | 0.70 |
| Obese | 16 (22.9 % ) | 0.75 (0.35-1.64) | 0.48 |
| **Educational qualification** |  |  |  |
| Middle school | 16 (22.9 %) | 1.00 |  |
| No qualification | 6 (8.6 %) | 1.55 (0.54-4.45) | 0.41 |
| Primary school | 31 (44.3 %) | 1.21 (0.58-2.49) | 0.61 |
| Diploma | 10 (14.3 %) | 1.37 (0.56-3.36) | 0.49 |
| Degree | 7 (10.0 %) | 2.40 (0.81-7.16) | 0.12 |
| **Physical activity** |  |  |  |
| No | 56 81.2 %) | 1.00 |  |
| Yes | 13 (18.8 %) | 1.30 (0.61-2.74) | 0.49 |
| **Smoking status** |  |  |  |
| Never-smoker | 13 (18.3 %) | 1.00 |  |
| Smoker | 8 (11.3 %) | 1.01 (0.32-3.17) | 0.99 |
| Ex-smoker | 50 (70.4 %) | 1.37 (0.59-3.17) | 0.46 |
| **Charlson index** |  |  |  |
| No comorbidity | 17 (24.3 %) | 1.00 |  |
| One comorbidity | 12 (17.1 %) | 1.21 (0.51-2.86) | 0.67 |
| Two or more comorbidities | 41 (58.6 %) | 4.17 (2.16-8.04) | <0.001 |
| **Deprivation index** |  |  |  |
| Very rich | 13 (19.1 %) | 1.00 |  |
| Rich | 5 (7.4 %) | 0.42 (0.10-1.68) | 0.22 |
| Medium | 11 (16.2 %) | 0.91 (0.32-2.59) | 0.86 |
| Deprived | 11 (16.2 %) | 0.75 (0.28-2.00) | 0.57 |
| Very deprived | 28 (41.2 %) | 1.03 (0.39-2.78) | 0.95 |
| **FEV1 (L), mean±sd** | 0.98 ± 0.41 | 0.26 (0.13-0.54) | <0.001 |
| **PaO2 (mmHg), mean±sd** | 73.19 ±13.90 | 1.01 (0.99-1.04) | 0.27 |

^a^HR adjusted for age, gender, BMI, educational qualification, physical activity, smoking status, Charlson index, deprivation index, FEV1, PaO2.
